# Birth Equity and Maternal Health Among Immigrant Communities in the United States: A Narrative Review

**DOI:** 10.3390/ijerph23060745

**Published:** 2026-06-02

**Authors:** Akanksha Anand, Ian Lindong, Sharon Barrett

**Affiliations:** School of Community Health and Policy, Morgan State University, Baltimore, MD 21251, USA

**Keywords:** birth equity, maternal health, immigrant communities, intersectionality, health equity, United States

## Abstract

Background: Immigrant communities and first-generation immigrants in the United States face persistent disparities in maternal health outcomes. These inequities are shaped by intersecting structural conditions, including socioeconomic exclusion, language barriers, cultural differences, and institutional constraints documented in prior research. Methods: This narrative review examined 28 peer-reviewed studies published between 2010 and 2024 that applied an intersectional framework to maternal health research focused on immigrant communities in the United States. Studies were identified through PubMed, Scopus, Web of Science, and Google Scholar. The review analyzed how each study conceptualized, designed, and interpreted maternal health in these populations. Results: Seven recurring themes were identified: barriers to and access to care; gaps in clinical guidance; limitations in health data and surveillance; immigration-related policy context; health system influences; intersectional vulnerability across subgroups; and the role of individual- and community-level supports. Conclusions: The literature highlights the importance of community-based strategies, Medicaid policy considerations, and culturally responsive care in addressing maternal health disparities among immigrant communities. Advancing birth equity will require coordinated efforts across healthcare systems, public health programs, and policy environments.

## 1. Introduction

Birth equity ensures that all individuals experience optimal birth outcomes by actively addressing racial and social inequality [1,2,3,4]. This concept is based on the principles of reproductive justice [1,2], which advocates for the human right to make autonomous choices about one’s body, including the right to have children, not having children, and raising children in secure and supportive communities. However, these frameworks arose in response to the legacy of conservative policies and reproductive oppression in the U.S., which have targeted immigrant women’s fertility and fostered distrust in medical systems [1,2,3,4]. Health equity is defined as the attainment of optimal health for everyone, ensuring that all individuals have fair and impartial opportunities to achieve their highest possible level of health, free from social or structural biases [3,4]. Maternal health inequities among immigrant populations have been widely documented in national datasets and peer-reviewed research, with multiple studies identifying elevated risks of severe maternal morbidity, barriers to prenatal care, and structural inequities in access to care across multiple U.S. contexts [5,6,7]. Reproductive justice emphasizes that racism and restricted bodily autonomy contribute to health disparities in marginalized communities. Achieving birth equity requires ensuring full bodily autonomy and access to comprehensive reproductive health services, including contraception, infertility treatment, and abortion care [8,9,10,11]. Thus, clinicians, researchers, and policymakers must actively advocate for these rights to improve maternal health outcomes.

The “Environmental Scan of Birth Equity and Quality Measurement” (BEAM report, December 2023) [12] offers a comprehensive overview of measurement approaches and thematic content in this field. We incorporated BEAM as an authoritative framing reference to position this review within the broader field consensus. While BEAM emphasizes frameworks and indicators for quality measurement, our review complements this by synthesizing intersectionality-informed empirical research focused on structural, social, and policy determinants shaping immigrant women’s maternal health experiences in the United States. This distinction helps clarify how measurement tools and experiential syntheses can be integrated to advance birth equity holistically. Unlike the BEAM report, which focuses on measurement frameworks and quality indicators, this review examines how intersectionality is operationalized across empirical maternal health studies involving immigrant communities.

Immigrant women face heightened risks of adverse maternal outcomes due to intersecting barriers, such as language access, cultural norms, socioeconomic exclusion, and discrimination [3,13,14,15,16]. These challenges often lead to delayed or avoided care, psychological distress, and limited access to culturally competent providers [17,18]. Prior research emphasizes the importance of culturally responsive, trauma-informed, and individualized care that reflects the diverse experiences of immigrant populations [19,20,21,22]. Strategies to promote equitable maternal care include multilingual services, diverse healthcare professionals, respect for cultural beliefs, and community engagement through collaboration with health workers, individuals, and trusted community leaders [10,19,23,24,25].

This narrative review highlights gaps in the existing research on the lack of representation of various immigrant women in maternal health studies in the United States [1,2]. Research indicates that structural inequities, including immigration enforcement policies and systemic barriers, contribute significantly to adverse maternal outcomes [3,4]. There is a significant gap related to their unmet needs. Consequently, it is essential to emphasize these underrepresented and understudied groups of immigrant women to achieve health equity. Immigrant women, especially those who are undocumented or come from marginalized racial groups, often face serious challenges when accessing quality maternal healthcare. Poverty, unstable housing, and lack of culturally sensitive care are just a few of the barriers that shape their experiences [1,2,3].

This narrative review synthesizes intersectionality-informed research examining maternal health disparities among immigrant communities in the United States. By integrating quantitative and qualitative evidence across healthcare and cultural contexts, the review identifies best practices, highlights complex structural influences, and informs policy- and practice-relevant strategies to improve maternal health outcomes. Across the literature, seven recurring themes emerged: barriers to care, gaps in clinical guidance, limitations in available data, immigration policy context, health system influences, community-based supports, and intersectional vulnerability.

### Public Health Relevance of Birth Equity for Immigrant Communities

Birth equity remains a core concern in public health because maternal health outcomes are shaped by the accumulation of social, structural, and policy conditions over the life course [1,2,3]. Prior public health scholarship has long emphasized the importance of examining these outcomes at the population level, particularly to identify patterned inequities and inform preventive, upstream approaches to intervention [12,26]. From this standpoint, differences in maternal health outcomes cannot be understood solely through biomedical risk factors but must also be situated within broader systems of resource allocation, institutional practice, and social organization [2,4].

Immigrant communities constitute a growing share of the U.S. population, rendering maternal health outcomes within these communities directly relevant to national public health priorities related to equity, quality of care, and population well-being [5,8,9]. Reviewing empirical evidence on how migration history, social position, and policy context interact to shape maternal health experiences offers practical value for public health surveillance, program design, and policy planning [9,10,11]. Consistent with population-level public health frameworks articulated by Ward [26] and Levisohn [7], this review approaches birth equity as a collective public health issue and contributes evidence to support the development of strategies aimed at improving maternal health outcomes across diverse immigrant communities.

## 2. Materials and Methods

### Design and Methodology

This study employed a narrative literature review to examine maternal health disparities among immigrant and first-generation women in the United States, with particular attention to studies applying an intersectionality framework. A narrative approach was selected to capture the breadth and complexity of research across diverse contexts and disciplines, enabling integration of theoretical perspectives, empirical findings, and policy discussions. An extensive search was conducted across four major academic databases—PubMed, Scopus, Web of Science, and Google Scholar—using combinations of the following terms: “maternal health disparities,” “immigrant women healthcare,” “birth equity,” “reproductive justice,” “intersectionality,” “racial disparities,” and “social determinants of maternal health.” The search was restricted to English-language peer-reviewed articles, government reports, and scholarly publications published between 2010 and 2024.

The review followed best practices for transparency and rigor in narrative synthesis, informed by Braun and Clarke’s reflexive thematic analysis framework [27]. Consistent with this approach, we engaged in a structured coding process that included data familiarization, code generation, theme development, and collaborative validation. A narrative thematic design was selected rather than a systematic review to allow for broader inclusion of empirical, qualitative, and policy-oriented scholarship. Studies were included if they explicitly referenced intersectionality as a guiding theoretical framework, focused on maternal or perinatal health outcomes, and examined populations that included racial/ethnic minorities and/or immigrant women in the United States. After screening titles, abstracts, and full texts, 28 publications met the inclusion criteria (Table A1). Among these studies, several explicitly applied intersectionality in their conceptual frameworks or analytic approaches, while others referenced intersectionality conceptually when discussing structural inequities affecting immigrant maternal health. A detailed summary of study aims, populations, and methodologies is provided in Table A2.

**Figure A1:**
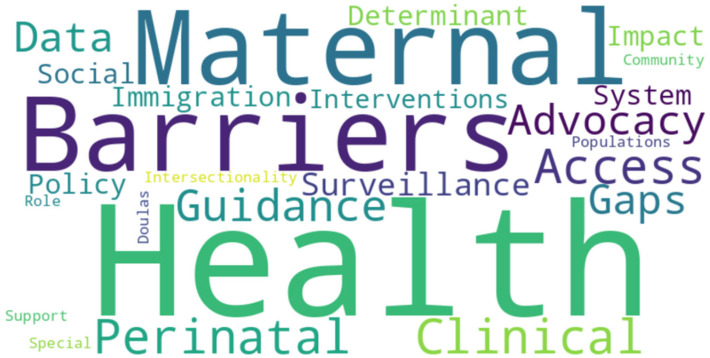
Thematic word cloud of literature included for narrative review.

Thematic analysis was used to identify recurring patterns, key concepts, and gaps within the literature. Researchers first engaged in independent data familiarization across included studies. Initial coding combined exploratory keyword scanning with manual validation to identify recurring concepts. Codes were grouped into preliminary thematic categories based on conceptual similarity and co-occurrence, and themes were refined through iterative discussion to ensure alignment with the review’s aims. Frequently recurring terms (e.g., barriers, doula, Medicaid, surveillance) are illustrated in Figure A1, with corresponding thematic interpretations detailed in Table A1 and Table A2.

To assess how intersectionality was operationalized across studies, we applied a structured evaluative framework examining four domains: (1) conceptual framing, (2) study design and sampling, (3) analytic methods, and (4) interpretation of findings, including acknowledgment of intersecting systems of oppression. A coding rubric was developed to guide consistent evaluation and is presented in Table A3. This framework enabled a systematic appraisal of how birth equity is addressed within existing scholarship and identified areas for more robust intersectionality-informed research. Although this review follows a narrative synthesis approach rather than a formal systematic review, we applied structured inclusion criteria and thematic coding procedures to enhance transparency and analytic rigor.

## 3. Results

### The Thematic Analysis Identified Seven Interrelated Themes That Characterize Structural, Policy, and Social Determinants Shaping Immigrant Maternal Health Experiences in the United States

(1) Barriers and access to care: Minority and immigrant women face layered systemic barriers to accessing quality maternal healthcare, including lack of insurance, language difficulties, discriminatory practices, and limited culturally competent services.
(2) Gaps in clinical guidance: Clinical guidelines for minoritized and immigrant women often overlook cultural nuances, failing to meet the specific needs of immigrant and marginalized communities. This gap is further widened by the lack of ongoing education for healthcare practitioners and an inadequate focus on care that prioritizes patients, collectively leading to more significant health disparities.
(3) Significant data gaps and surveillance concerns remain in addressing maternal health disparities. In particular, without detailed disaggregated data by race, ethnicity, and immigration status, the data-gathering process for analysis becomes more complicated. These recognized gaps are evident in datasets that reveal limitations, hindering the accurate monitoring of maternal health patterns and trends by delaying targeted interventions for minoritized populations.
(4) Immigration is a key social determinant of health that significantly influences birth equity among immigrant women. Immigrant women often face unique challenges that can adversely affect their reproductive health outcomes including limited access to healthcare services, language barriers, cultural differences in healthcare practices, and socioeconomic disparities. These factors can lead to inadequate prenatal care, increased stress during pregnancy, and high rates of complications during childbirth. Additionally, immigration status may influence health-seeking behaviors, with undocumented immigrants potentially avoiding medical care because of fear of deportation. The intersection of immigration with other social determinants, such as race, ethnicity, and socioeconomic status, further compounds health inequities, resulting in disproportionately poor birth outcomes among immigrant women compared with their native-born counterparts.
(5) Policies and system-level effects play a crucial role in shaping birth outcomes among minority immigrant women. These overarching structures influence access to health care, social support, and economic resources, which are vital determinants of maternal and infant health. Systemic barriers such as language difficulties, cultural misunderstandings, and discriminatory practices can significantly hinder the ability of minority immigrant women to receive adequate prenatal care and support. In addition, immigration policies may create stress and uncertainty, potentially affecting pregnancy outcomes. Addressing these challenges requires comprehensive policy reforms to promote cultural competence in healthcare, improve language accessibility, and ensure equitable access to resources. By implementing inclusive policies and dismantling systemic barriers, policymakers can reduce disparities and improve birth outcomes among minority immigrant women.
(6) Community-Based Supports and Culturally Responsive Care. Community-based and culturally responsive support systems play an important role in improving maternal health outcomes among immigrant women. Doulas—trained professionals who provide emotional, physical, and informational support before, during, and after childbirth—play a crucial role in enhancing birth experiences and maternal satisfaction. Research indicates that the presence of a doula during labor and delivery is associated with reduced cesarean delivery rates, decreased use of pain medication, shorter labor durations, improved breastfeeding initiation, and higher levels of maternal satisfaction. These outcomes are achieved through continuous emotional support, comfort measures, and advocating for mothers’ preferences during childbirth. Doulas also help bridge communication gaps between healthcare providers and immigrant families by facilitating culturally informed care and supporting shared decision making.
  Beyond doula support, broader community-based interventions—including community health workers, culturally responsive perinatal programs, and partnerships with local organizations—have shown promise in improving maternal health outcomes among immigrant populations. Programs that integrate cultural humility, trauma-informed care, and community engagement can help address structural barriers such as language differences, healthcare navigation challenges, and limited access to resources. Community support networks—including family members, peer groups, and local organizations—also provide practical and emotional assistance that can reduce stress, improve access to services, strengthen social connections, and enhance maternal confidence during pregnancy and the postpartum period. By incorporating cultural competence and community engagement into maternal care programs, healthcare systems can better address immigrant women’s needs and contribute to advancing birth equity. Similar to findings in clinical trial contexts, community-engaged strategies have also demonstrated effectiveness in improving inclusion and trust among diverse populations [28].
(7) Heightened risks from intersecting identities and intersectional vulnerability: The intersection of racial background, immigration status, and socioeconomic factors exacerbates vulnerability to adverse maternal and infant health outcomes. Individuals with multiple marginalized identities encounter compounded challenges in accessing health care services. These individuals are less likely to receive timely prenatal care because of barriers such as linguistic difficulties, lack of health insurance coverage, and limited awareness of available resources. Furthermore, they are more likely to experience complications during childbirth, potentially attributable to stress, inadequate nutrition, and delayed medical intervention. This intersectionality of risk factors underscores the necessity for targeted interventions and policies that address the unique challenges faced by individuals with multiple marginalized identities in the context of maternal healthcare.

Consistent keyword patterns across studies supported these seven themes. The characteristics of the included studies, including aims, populations, and methodologies, are summarized in Table A2 (Appendix A). The thematic word cloud visually represents term frequency (Figure A1). These findings affirm the existing evidence that structural inequities continue to undermine reproductive justice for marginalized women in the United States. Equity challenges in maternal health reflect broader patterns across medical specialties. Similar systemic disparities have been documented in urologic oncology, emphasizing the need for structural interventions in both access and quality of care [29]. Reproductive justice [30,31], as a framework, is particularly relevant, as it emphasizes autonomy, intersectionality, and community well-being. The compounded effect of social determinants of health, such as poverty, education, housing, and immigration policy, contributes significantly to poor maternal outcomes.

## 4. Barriers and Access Affecting Immigrant Women and First-Generation Immigrants

Immigration status significantly influences health outcomes and is recognized as a key social determinant of health [1]. Internationally, ecological systems theory has been used to understand maternal health disparities in ethnic minority rural populations, as seen in China [32]. This highlights the value of multilevel frameworks in analyzing how place, policy, and identity intersect to shape maternal health. Despite progress in adopting evidence-based practices in maternal healthcare, immigrant mothers continue to face inequitable access to essential services due to pervasive social, structural, economic, and racial injustices [2,3]. Nonetheless, research on health policies frequently neglects the effects of immigrant exclusion in efforts to advance perinatal health equity [2]. The American College of Obstetricians and Gynecologists (ACOG) [8] issued committee recommendations highlighting the significance of health and well-being for everyone seeking obstetric and gynecological services, irrespective of their immigration status. The statement pointed out that immigrants may encounter unique healthcare challenges due to their circumstances, such as injuries or trauma experienced during migration, exposure to environmental hazards, and advanced stages of certain illnesses [8]. Nonetheless, the practical application of this approach is hindered by differing state laws that frequently limit insurance coverage for undocumented immigrants and those who have obtained legal documentation within the past five years [9].

## 5. Intersection of Race, Immigration Status, and Socioeconomic Factors

Maternal health outcomes for mothers born outside the United States are significantly influenced by both their country of origin and their location within the U.S. This results in different levels of risk for adverse maternal and neonatal outcomes, shaped by social health determinants, such as education, neighborhood conditions after resettlement, income, and poverty [5,10]. Although immigrants represent only 14% of the overall U.S. population, they account for nearly a quarter (23%) of all newborns in the nation [11,33]. The U.S. has the highest maternal mortality rate among developed nations, and this rate continues to rise [5,34]. The Affordable Care and Patient Protection Act (ACA) established a policy framework to strengthen protection against discrimination for people with limited English proficiency in U.S. healthcare settings, aiming to address long-standing disparities in healthcare access and quality among linguistically diverse populations. Despite this legislation, patients with language barriers continue to face barriers in maternal care and communication, indicating persistent implementation challenges.

Almost 70% of maternal deaths are preventable [35], and immigrant women are more vulnerable [5,13,14,36]. In addition, these women face considerable barriers when trying to access perinatal services [2,14,37]. These services are important for maintaining maternal and family health across generations [38,39]. Not only does it enhance health, but it also aids in preparing immigrant women during the period before childbirth, while in the early stages of pregnancy, it assists in proactive problem mitigation and early diagnosis of problems such as anemia, hypertensive disorders of pregnancy, and infections. Additionally, it correlates with neonatal and low birth weight mortality [8,35].

Accessing high-quality, culturally competent perinatal care poses challenges for ethnic minorities both globally and in the U.S. [23,40]. For immigrant women, barriers include a lack of insurance, language difficulties, insufficient understanding of the healthcare system’s complexities, unavailability of interpreters for certain dialects, transportation challenges, distrust of the medical system, disruption of traditional support networks, conflicts with healthcare providers, and experiences of discrimination [15,19,41]. Additionally, an insufficient number of providers who exhibit cultural competence, religious respect, and trauma-informed training further undermines the quality of perinatal services [16,20,21,42]. These barriers pose significant risks to maternal health and contribute to increased morbidity and mortality. A holistic approach requires attention to social determinants of health and structural inequities that influence policy and practice [14,24].

Additionally, some socioeconomic factors affect pregnancy-related issues among immigrants. Undocumented immigrants tend to have informal, uncontracted, and non-legally bound employment [43,44]. Because reporting workplace grievances or claiming maternity benefits places undocumented immigrants in an at-risk frame, they become socially constructed and vulnerable to exploitation and fundamental inequality discrimination. This leads to structural discrimination, which includes peripheral barriers that restrict access to perinatal care, consistent with global findings on migrant workers’ precarious employment conditions [45]. Even documented immigrants struggle with low-paying, high-risk-saturated employment due to a lack of experience or qualifications. Many are paradoxically overqualified for paying and unrecognized work stalled by their foreign credentials in the U.S., which adversely impacts immigrant families and the broader economy [44].

## 6. Impact of Challenges on Maternal and Infant Health

Adverse Maternal and Infant Health Outcomes: Immigrant women often face significant barriers—including low-wage employment, unrecognized foreign credentials, and limited access to culturally responsive care—that heighten their vulnerability during pregnancy [22,44]. These structural and psychosocial challenges contribute to delayed prenatal care, elevated stress levels, and an increased risk of adverse maternal and infant health outcomes [17,43]. Some of these structural vulnerabilities have also been documented in adolescent maternal populations, underscoring the broader role of socioeconomic and healthcare access barriers in shaping maternal risk [38,39].

Teen pregnancy has been associated with elevated risks of adverse maternal and infant outcomes, including pregnancy-induced hypertension, anemia, preeclampsia, preterm birth, and low birth weight [14,23,38,39,40]. However, evidence suggests that these risks are often mediated by underlying structural determinants—such as socioeconomic disadvantage, limited access to prenatal care, insurance instability, and educational disruption—rather than maternal age alone. In contexts where healthcare access is constrained, including among marginalized or immigrant communities, these structural barriers may compound vulnerability and contribute to higher rates of cesarean delivery, postpartum complications, and neonatal morbidity [34,38,43].

Beyond clinical outcomes, adolescent pregnancy can intersect with social exclusion, educational discontinuity, and economic instability, reinforcing long-term inequities [16,24,31]. Importantly, these psychosocial and structural stressors are not uniformly distributed; they disproportionately affect populations experiencing poverty, immigration-related exclusion, or limited healthcare access. Thus, when adolescent pregnancy occurs within structurally marginalized communities, it reflects broader systemic conditions rather than isolated individual risk factors [44,46].

## 7. Strategies for Improving Healthcare Access and Quality

Community-based interventions play an important role in reducing disparities in maternal and perinatal health among immigrant communities [47,48]. These initiatives involve collaborative partnerships between healthcare providers, community organizations, and local leaders to introduce tailored maternal support programs. By leveraging community resources and local knowledge, these interventions improve care navigation, reduce barriers to prenatal and postpartum services, and expand access to culturally responsive maternal healthcare [20,21]. Community-based approaches are particularly effective in utilizing existing social networks and cultural norms to create sustainable, culturally sensitive solutions that improve maternal health outcomes [16].

A key feature of effective maternal health interventions is their emphasis on cultural competence [20]. Involving community members in planning and implementation ensures that programs address the linguistic, cultural, and socioeconomic factors influencing pregnancy and childbirth experiences. This approach helps overcome barriers such as language differences, mistrust of healthcare systems, and limited familiarity with available services. Capacity-building strategies—such as training community health workers or culturally matched doulas—expand the reach of maternal support services while fostering trust and long-term sustainability within communities.

Community-based interventions also recognize the influence of social determinants on maternal outcomes. Programs may integrate support related to housing stability, employment conditions, education, and social support, all of which shape pregnancy experiences and postpartum recovery [23]. By addressing these broader structural factors, such initiatives contribute to a more holistic and sustainable model of maternal care. Importantly, community-integrated models can engage women who might otherwise face barriers to accessing traditional prenatal or postpartum services due to transportation challenges, documentation concerns, or limited awareness of available resources [18].

Support networks and advocacy groups further contribute to improving maternal healthcare access among immigrant women and first-generation populations. These organizations provide language assistance, childbirth education, navigation support, and peer-based guidance to help women understand their rights and access services [13,38]. Advocacy efforts promote inclusive policy reforms and cultural competence within maternity care systems. By amplifying the voices of immigrant communities and fostering collaboration among providers, policymakers, and community stakeholders, these networks play a critical role in advancing birth equity.

Teenage pregnancy among immigrant and minority youth highlights the need for culturally responsive reproductive education and early maternal health support services [6,38]. Literature also underscores the ongoing lack of culturally competent and trauma-informed maternity care in many healthcare settings [20,21,49]. Although professional organizations have issued statements supporting inclusive care (e.g., ACOG) [8], implementation remains inconsistent across states due to Medicaid exclusions and immigration-related policy variability [2,50]. There is a continued need for longitudinal and community-based participatory research that centers immigrant mothers’ lived experiences [14,19,51]. Future research should evaluate the long-term impact of community-led maternal interventions and explore digital health innovations tailored to immigrant populations [22,47].

### 7.1. Community-Based Practice Example: Community Doula Program for Immigrant Women in Sweden

In Sweden, a community-based bilingual doula program has been implemented to support immigrant women during labor and childbirth. The program was designed to address challenges frequently reported by immigrant women, including linguistically accessible services, cultural differences, and limited family or social support during the perinatal period [16]. Doulas who share linguistic and cultural backgrounds with the women they support play a critical role in bridging these gaps by providing continuous emotional, informational, and physical support. In addition, doulas facilitate communication between women and healthcare providers, helping ensure that women’s needs, preferences, and concerns are understood and respected during childbirth [22,52].

### 7.2. Real-World Implications

Evidence from qualitative and mixed-methods evaluations indicates that participation in the program is associated with improved birth experiences among immigrant women. Women reported feeling more involved in decision making, experiencing reduced anxiety during labor, and gaining a greater sense of autonomy and reassurance through continuous doula support [22]. The presence of doulas was particularly valued in situations where women felt fearful or isolated, underscoring the importance of sustained, culturally responsive support during childbirth [22,53]. Healthcare providers also reported benefits, noting improved communication, smoother care coordination, and enhanced cultural understanding within maternity care settings [53].

### 7.3. Practitioner Perspective

As one midwife involved in the program noted:

The presence of community-based individuals has transformed the way we provide care to immigrant women. They not only bridge language gaps but also bring cultural understanding that is invaluable for creating a supportive and respectful birthing environment [53].

### 7.4. U.S. Potential for Strategic Adaptation

The Swedish community doula model offers transferable insights for the U.S. healthcare context, where immigrant women face similar challenges related to language access, cultural concordance, and limited support networks. Pilot doula programs implemented in states such as New York and California have demonstrated the feasibility of integrating culturally matched support workers into maternity care settings. Expanding such initiatives nationally—particularly through Medicaid reimbursement and formal integration into maternity care teams—may represent a viable strategy for improving maternal care experiences and advancing birth equity among immigrant communities in the United States.

## 8. Policy Recommendations

These findings collectively demonstrate that maternal health disparities among immigrant populations are shaped by interacting structural determinants operating across healthcare systems, policy environments, and community contexts. Expanding Medicaid is an important strategy for addressing persistent disparities in maternal and infant health among immigrant women and first-generation populations in the United States [6,50,54]. Structural barriers—including immigration-related eligibility restrictions and gaps in culturally responsive care—continue to limit equitable access to prenatal and postpartum services. Evidence indicates that Medicaid expansion is associated with improved birth equity outcomes, particularly in marginalized communities, by increasing access to essential maternal health services [6].

Federal and state policy decisions directly shape healthcare accessibility through insurance eligibility, financial assistance programs, and postpartum coverage continuity. Expanding publicly funded insurance options can mitigate financial barriers to care, especially for immigrant populations excluded from traditional coverage pathways [6,50,54]. For example, California’s expansion of Medicaid to include income-eligible individuals regardless of immigration status has been associated with improved perinatal access and continuity of care [50]. Similarly, New York’s Medicaid-supported doula pilot programs demonstrate promising maternal outcomes and illustrate scalable, community-integrated approaches [54].

At the community level, investments in local health infrastructure further strengthen access and quality. Supporting community health organizations, expanding clinic capacity, and integrating telehealth services can improve reach in underserved areas. Coordinated federal, state, and community-level efforts are therefore essential to advancing maternal health equity and reducing persistent disparities.

## 9. Limitations

This review has several limitations that should be acknowledged. First, as a narrative review, the study did not include a formal meta-analysis or statistical synthesis of findings across studies. The articles included also differed in research design, sample populations, methodologies, and outcome measures, making direct comparisons across studies challenging. Additionally, variations in immigration classifications and reporting definitions across studies may have limited the comparability of findings. The review was also limited to English-language publications, which may have excluded relevant international research published in other languages. It is also possible that publication bias influenced the available literature, as studies reporting significant findings are more likely to appear in peer-reviewed journals and major academic databases. Furthermore, the findings discussed in this review reflect patterns identified within the existing literature and may not fully capture the experiences of all immigrant populations or healthcare settings in the United States. As a narrative synthesis, this review prioritizes conceptual integration and thematic interpretation rather than exhaustive systematic screening or pooled statistical estimation. Despite these limitations, the review provides an important overview of current evidence related to birth equity, maternal health disparities, and the experiences of immigrant communities in the United States.

## 10. Future Contributions

Future research should continue to examine maternal health disparities among immigrant communities through longitudinal, mixed-methods, and community-based participatory approaches that center women’s lived experiences across the prenatal, perinatal, and postpartum continuum [14,18,51]. Empirical studies are needed to better understand how immigration policy environments, Medicaid eligibility criteria, and culturally concordant care models interact to shape maternal outcomes. Evaluating the long-term effectiveness of community-led interventions—including doula integration and community health worker programs—will be critical for identifying scalable strategies that reduce structural inequities.

Strengthening national and state-level data systems through disaggregated reporting by immigration status, race/ethnicity, and language preference can improve surveillance precision and inform targeted interventions. Implementation science frameworks may further clarify how culturally responsive maternity care models can be adapted across diverse healthcare systems and policy contexts. Sustained collaboration among researchers, healthcare practitioners, policymakers, and community organizations remains essential to translating empirical findings into actionable reforms that advance birth equity for immigrant populations.

## 11. Conclusions

Birth equity remains an important public health priority, with persistent disparities across racial, ethnic, and socioeconomic groups. Key factors include systemic racism, limited access to quality prenatal care, and the social determinants of health. Recent research emphasizes culturally competent care, community-based interventions, and addressing implicit bias to improve outcomes in marginalized populations [7,47,55]. Ongoing efforts to promote birth equity are crucial for reducing maternal and infant mortality rates, improving population health, and addressing social injustices. These efforts may improve maternal and infant health outcomes, reduced healthcare costs, and enhanced community wellbeing. Recent state-level initiatives, such as California’s expansion of Medi-Cal regardless of immigration status and New York’s Medicaid-funded doula pilot program, offer practical models for improving access and equity in maternal care [50,54]. Future research should focus on innovative interventions such as integrating community health workers and implementing technology-based solutions [17,49]. Policy efforts should prioritize funding for maternal health initiatives, expand Medicaid coverage for postpartum care, and implement standardized training programs on cultural competence and implicit bias [30,31]. Interdisciplinary collaboration should be encouraged to develop comprehensive strategies for promoting birth equity.

**Figure 1:**
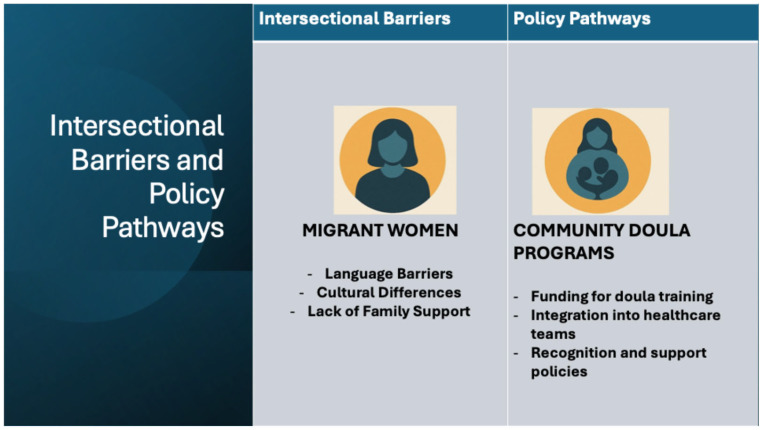
Conceptual framework illustrating intersectional barriers affecting immigrant maternal health and policy pathways for advancing birth equity.

As shown in Figure 1, immigrant women face overlapping challenges, including language barriers, cultural differences, and limited familial support during the perinatal period. The figure illustrates how policy pathways—such as funding for doula training, integration of doulas into maternity care teams, and formal recognition of culturally matched support roles—can address these intersectional barriers and support the sustainability of community-based maternal care programs [47,48,51]. Together, these strategies demonstrate how community-integrated models can help bridge equity gaps in maternal healthcare.

Community-based doula programs have demonstrated their potential to significantly improve childbirth experiences of migrant women by providing culturally responsive care and bridging critical gaps in communication and support. These programs not only benefit women but also enhance the capacity of healthcare providers to deliver high-quality, culturally competent care. Further research and policy support are essential to expand and sustain these valuable programs.

To strengthen these efforts, community engagement must extend beyond outreach to include the identification, training, and formal support of community-based volunteers, including bilingual doulas and community health workers (CHWs). These individuals serve as trusted liaisons between healthcare systems and immigrant families. Supporting their certification, leadership pathways, and fair compensation can sustain long-term impact. Local partnerships—with faith-based groups, cultural organizations, and public health departments—are essential to build and maintain this volunteer capacity [18].

## Data Availability

No new data were collected or analyzed in this study. Data sharing is not applicable to this article.

## Appendix A

**Table A1:** Thematic analysis of the literature included in the narrative review.

Theme	Number of Authors	Authors
Barriers to Maternal and Perinatal Health Access	9	Behboudi-Gandevani et al. (2022) [56], Bhasin (2022) [36], Fox et al. (2023) [2], Heslehurst et al. (2018) [38], Merry et al. (2023) [19], Small et al. (2014) [41], Toh & Shorey (2023) [57], U.S. GAO (2021) [13], van den Akker & van Roosmalen (2016) [3]
Clinical Guidance and Advocacy	5	ACOG (2025) [8], Benza & Liamputtong (2014) [20], Fair et al. (2020) [21], Fitzharris, et al. (2025) [58], Mehta et al. (2021) [35]
Data Gaps and Surveillance	5	CMS (2022) [4], Hoyert (2023) [5], Livingston (2016) [10], Livingston (2016) [11], U.S. Census Bureau (n.d.) [33]
Immigration as a Social Determinant of Health	7	Bhasin (2022) [36], Castañeda et al. (2015) [1], Crookes et al. (2022) [9], Fox et al. (2023) [2], Korinek & Smith (2011) [42], Fitzharris et al. (2025) [58], Steenland et al. (2023) [15]
Impact of Policy and Health System Interventions	8	CMS (2022) [4], Commonwealth Fund (2020) [34], ILO (2023) [45], Mosley et al. (2021) [48], Pillai & Artiga (2023) [44], Swartz et al. (2017) [39], Holcomb et al. (2025) [23], WHO (2021) [43]
Intersectionality and Special Populations	3	Fortuna et al. (2019) [16], Maru et al. (2021) [59], Mehta (2021) [35]
Role of Doulas and Community Support	5	Helena (2021) [52], Khaw (2022) [47], Khaw (2023) [51], Purandare (2024) [22], Schytt (2021) [53]

**Table A2:** Birth Health Equity and Immigration Literature Table.

First Author (Publication Year)	Type Journal Article	Stated Aim	Populations	Before or After ACA	Total Sample Size	Data Collection Method
Castañeda et al. (2015) [1]	Review Article	To explore how immigration acts as a social determinant of health and affects healthcare access and health outcomes.	Immigrants in the U.S.	After ACA	N/A (Review article)	Literature review and policy analysis
Fox et al. (2023) [2]	Empirical Research Article	To examine the impact of Medicaid immigrant exclusions on maternal health care access across the reproductive-perinatal continuum.	Immigrant women of reproductive age	After ACA	Sample size not explicitly stated	Quantitative analysis using survey and administrative data
Van den Akker & van Roosmalen (2016) [3]	Review Article	To review maternal mortality and severe maternal morbidity from a migration perspective.	Migrant and immigrant women	Both before and after ACA	N/A (Review article)	Literature review
CMS (2022) [4]	Government Report	To outline CMS’s 10-year strategic plan to advance health equity by improving access, outcomes, and data.	Underserved populations including racial/ethnic minorities, LGBTQ+, and rural communities	After ACA	N/A (Policy document)	Policy framework and administrative data analysis
ACOG (2025) [8]	Committee Opinion/Clinical Guidance	To provide recommendations and highlight issues regarding healthcare access for immigrant women.	Immigrant women in the U.S.	After ACA	N/A	Expert opinion and clinical guidelines
Crookes et al. (2022) [9]	Systematic Review	To examine how immigrant-related policies impact the health outcomes of Latinx adults in the U.S.	Latinx immigrant adults	After ACA	N/A (Systematic review)	Systematic review of peer-reviewed studies
Livingston (2016a) [10]	Research Report	To provide demographic data on immigrants in the U.S., including age, education, employment, and region of origin.	Immigrants in the U.S.	After ACA	N/A (Population-level data)	Secondary data analysis using U.S. Census and national datasets
Livingston (2016b) [11]	Research Report	To examine the contribution of immigrants to U.S. birth rates and fertility trends.	Immigrant families and childbearing-age women	After ACA	N/A (Population-level data)	Secondary data analysis using birth and census data
U.S. Census Bureau (2024) [33]	Government Report	To describe demographic and housing characteristics of the foreign-born population in the United States.	Foreign-born individuals in the U.S.	After ACA	Large-scale national data (exact number not specified)	Secondary data analysis using American Community Survey and Census data
Commonwealth Fund (2020) [34]	Policy Report	To highlight the growing crisis of maternal mortality in the U.S. and identify contributing factors.	Pregnant and postpartum women in the U.S.	After ACA	N/A	Policy analysis and secondary data review
Hoyert (2023) [5]	Data Brief	To report maternal mortality rates and identify trends and predictors over time in the U.S.	Women who died due to maternal causes in the U.S.	After ACA	National data; exact size varies by year	Analysis of death certificate data
Mehta et al. (2021) [35]	Policy Statement	To issue a call to action for improving maternal health and saving mothers’ lives.	Pregnant and postpartum women, particularly underserved groups	After ACA	N/A	Policy synthesis and expert recommendations
Bhasin (2022) [36]	Legal Commentary	To explore how immigration enforcement affects undocumented parents’ rights to raise their children in a safe and healthy environment.	Undocumented immigrant families	After ACA	N/A	Legal case review and policy analysis
U.S. GAO (2021) [13]	Government Report	To investigate the barriers immigrant women face in accessing maternal healthcare in the U.S.	Immigrant women	After ACA	N/A (Government inquiry)	Interviews, case studies, and policy analysis
Behboudi-Gandevani et al. (2022) [56]	Systematic Review	To evaluate the impact of maternal immigration status on perinatal care utilization.	Immigrant women during the perinatal period	After ACA	N/A (Systematic review)	Systematic review of observational studies
Maru et al. (2021) [59]	Empirical Research Article	To assess maternal health outcomes among immigrant women and advocate for targeted interventions.	Immigrant women	After ACA	Not specified in abstract	Quantitative analysis using health records and surveys
Heslehurst et al. (2018) [38]	Literature Review	To examine the role of health care professionals in improving perinatal outcomes for women from diverse backgrounds.	Women from ethnically diverse backgrounds	After ACA	N/A (Review article)	Literature review
Swartz et al. (2017) [39]	Empirical Research Article	To evaluate the effects of expanding prenatal care to unauthorized immigrant women on infant health outcomes.	Unauthorized immigrant women and their infants	After ACA	Large sample size (Oregon birth records)	Quasi-experimental design using birth outcomes data
Toh & Shorey (2023) [57]	Qualitative Study	To explore perinatal care experiences among ethnic minorities.	Ethnic women from communities of color	After ACA	Varied (qualitative interviews)	In-depth interviews and thematic analysis
Holcomb et al. (2025) [23]	Empirical Research Article	To examine how social determinants of health shape maternal care access and outcomes.	Women receiving maternity care in diverse communities	After ACA	Not specified	Survey and community-based participatory research
Merry et al. (2023) [19]	Qualitative Study	To understand barriers to perinatal care for immigrant women from healthcare providers’ perspectives.	Immigrant women, healthcare providers	After ACA	N/A (qualitative)	Interviews and thematic analysis
Small et al. (2014) [41]	Qualitative Study	To explore maternity care access barriers among immigrant women in Australia.	Immigrant women	Before ACA	N/A (qualitative)	Qualitative interviews and focus groups
Steenland et al. (2023) [15]	National Study	To investigate healthcare access barriers for undocumented immigrants in the U.S.	Undocumented immigrants	After ACA	National sample; not specified	Quantitative survey
Benza & Liamputtong (2014) [20]	Conceptual Article	To discuss cultural competence and humility in healthcare delivery.	Healthcare providers and diverse patients	Before ACA	N/A	Conceptual framework analysis
Fair et al. (2020) [21]	Clinical Resource	To provide trauma-informed care guidance for women’s health care providers.	Women receiving reproductive and general health care	After ACA	N/A	Resource synthesis and expert recommendations
Fortuna et al. (2019) [16]	Empirical Research Article	To assess how cultural and linguistic barriers affect mental health treatment of immigrant women.	Immigrant women receiving mental health services	After ACA	Not specified	Quantitative analysis
Korinek & Smith (2011) [42]	Empirical Research Article	To examine the relationship between immigration legal status and prenatal care access.	Immigrant and racially ethnic women	Before ACA	Not specified	Survey data analysis
Fitzharris (2025) [58]	Policy Analysis	To examine barriers and facilitators affecting asylum seekers’ and refugees’ access to non-hospital-based healthcare services.	Immigrant women in the U.S.	After ACA	N/A	Policy review and analysis
Mosley et al. (2021) [48]	Empirical Research Article	To evaluate birth outcomes from a community-based pregnancy support program for refugee women in Georgia.	Refugee women in Georgia, U.S.	After ACA	Not specified	Program evaluation and outcome analysis
WHO (2021) [43]	Global Report	To assess health concerns and priorities of migrant workers globally.	Migrant workers worldwide	After ACA	N/A	Global surveillance and policy analysis
ILO (2023) [45]	Global Policy Report	To examine global labor rights and protections for migrant workers.	Migrant workers globally	After ACA	N/A	Policy analysis and international labor data
Pillai & Artiga (2023) [44]	Policy Report	To assess the economic impact of immigration on U.S. families and communities.	Immigrant families and U.S. communities	After ACA	N/A	Policy analysis and secondary economic data
Global and Non-US						
Purandare et al. (2024) [22]	Mixed Methods Study	To explore migrant women’s experiences with community-based doula support during childbirth in Sweden.	Migrant women in Sweden	N/A (Sweden)	Not specified	Mixed methods (survey and interviews)
Schytt et al. (2021) [53]	Qualitative Study	To examine midwives’ and obstetricians’ experiences with community-based bilingual doulas.	Healthcare providers in Sweden	N/A (Sweden)	Not specified	Qualitative interviews
Helena et al. (2021) [52]	Qualitative Study	To understand doulas’ perspectives on their work supporting migrant women in Sweden.	Community-based bilingual doulas	N/A (Sweden)	Not specified	Interviews and thematic analysis
Khaw et al. (2022) [47]	Systematic Review	To synthesize evidence on community-based doulas for migrant and refugee women.	Migrant and refugee women	N/A (Global)	N/A (Systematic review)	Mixed-method systematic review
Khaw et al. (2023) [51]	Qualitative Study	To describe community-based doulas’ roles in providing culturally responsive care to migrant women.	Migrant women in Australia	N/A (Australia)	Not specified	Qualitative interviews

**Table A3:** Rubric for Assessing Intersectionality Application in Included Studies. To assess the degree to which intersectionality was operationalized in each study, we applied a four-domain rubric. Each domain was evaluated for explicitness, integration, and depth of application based on the following criteria.

Domain	Evaluation Criteria	Indicators of Strong Application
1. Conceptual Framing	Did the study define intersectionality and integrate it into the theoretical framework?	Explicit reference to intersectionality theory; discussion of power, identity, systems of oppression.
2. Study Design and Sampling	Was intersectionality reflected in study population selection and sampling strategy?	Inclusion of multiple marginalized identities; sampling aimed at intersectional experiences.
3. Analytic Methods	Were methods suited to exploring intersectional complexity?	Use of stratified analyses, mixed methods, or thematic coding aligned with intersectional theory.
4. Interpretation and Findings	Did interpretation address how overlapping systems shape experiences?	Discussion of racism, classism, xenophobia, or policy impacts as interlocking forces.
